# Transmission of SARS-CoV-2 between ferrets in presence of pre-existing immunity

**DOI:** 10.1128/jvi.01566-25

**Published:** 2025-11-04

**Authors:** Chong Wang, Lei Shuai, Gongxun Zhong, Zhiyuan Wen, Renqiang Liu, Qilong Liu, Jinliang Wang, Jinying Ge, Xianfeng Zhang, Yuntao Guan, Xijun He, Zhigao Bu

**Affiliations:** 1 State Key Laboratory for Animal Disease Control and Prevention, Harbin Veterinary Research Institute, Chinese Academy of Agricultural Sciences 111613 , Harbin, People's Republic of China; 2 National High Containment Laboratory for Animal Diseases Control and Prevention, Harbin, People's Republic of China; 3 Jiangsu Co-innovation Center for Prevention and Control of Important Animal Infectious Diseases and Zoonoses, Yangzhou, People's Republic of China; University of North Carolina at Chapel Hill, Chapel Hill, North Carolina, USA

**Keywords:** SARS-CoV-2, transmission, pre-existing immunity

## Abstract

Severe acute respiratory syndrome coronavirus 2 (SARS-CoV-2), the etiological agent of coronavirus disease 2019 (COVID-19), is characterized by its high contagiousness. The COVID-19 pandemic has exerted profound impacts on human society. The persistent circulation of SARS-CoV-2 in human populations continues to pose re-exposure risks for both vaccinated individuals and those with prior natural infection. Against this epidemiological background, there is an urgent need to characterize the transmission dynamics of SARS-CoV-2 in the context of pre-existing immunity. Using a ferret infection model, this study systematically addresses critical scientific questions, including viral transmission efficiency, temporal patterns of transmissibility, and the ability of pre-existing immunity to mitigate reinfection and viral shedding. The findings provide robust experimental evidence for elucidating the transmission mechanisms of SARS-CoV-2 and offer scientific insights to inform the rational design of optimized antiviral strategies.

**IMPORTANCE:**

In this study, a ferret infection model was used to systematically investigate SARS-CoV-2’s *in vivo* transmission dynamics and modes with pre-existing immunity. By characterizing viral transmission efficiency, temporal shedding patterns, and immunity’s role in mitigating re-infection severity and dissemination, it provides direct evidence for understanding SARS-CoV-2 spread in immune-exposed hosts. It quantifies how humoral immunity modulates viral load (primary/secondary infections) and ferret contact transmission. Findings establish a key SARS-CoV-2 transmission framework—pre-existing immunity shortens shedding, reduces secondary attack rates but retains residual transmissibility—fill knowledge gaps, guide vaccine/herd immunity/public health measures, and lay a foundation for predicting real-world transmission and antiviral policies via integrated endpoints.

## INTRODUCTION

Severe acute respiratory syndrome coronavirus 2 (SARS-CoV-2), the causative agent of coronavirus disease 2019 (COVID-19), displays high contagiousness in humans. As a catastrophic pandemic, COVID-19 has significantly impacted human society, highlighting the urgency of deciphering its epidemiological dynamics. With the persistent circulation of SARS-CoV-2 in global populations, there is a critical need to investigate its transmission patterns in the context of pre-existing immunity (e.g., from vaccination or prior infection).

Animal infection models are indispensable for characterizing the infection biology, pathogenesis, and immunology of SARS-CoV-2. To date, numerous animal models have been documented, including non-human primates (NHPs), mice, golden hamsters, and ferrets, among others (1–7). Among these, ferrets are widely used in studies of respiratory infectious disease transmission and pathogenesis—such as influenza viruses and certain paramyxoviruses—due to their relatively small size and anatomical/physiological similarities to humans (8–10). For SARS-CoV-2 research, ferrets have proven suitable: our group and others have previously demonstrated that SARS-CoV-2 can infect ferrets, replicate in the upper respiratory tract, and induce pulmonary lesions (2, 11). Viral shedding and droplet-mediated transmission have also been observed in these models, making ferrets well-suited for investigating SARS-CoV-2 transmission dynamics and vaccine-induced protection under controlled experimental conditions (12).

SARS-CoV-2’s high transmissibility, evidenced by the predominance of cluster infections in COVID-19 cases, underscores close contact as a primary transmission route. With the ongoing global spread of the virus, urgent interventions such as vaccines and antiviral drugs remain critical. As SARS-CoV-2 is expected to become an endemic pathogen in humans—similar to influenza virus—vaccinated individuals face continuous risk of re-exposure. Key questions regarding vaccination and re-infection must be addressed: How efficient and rapid is viral transmission? Can pre-existing immunity in convalescent individuals or vaccine recipients prevent re-infection and viral shedding? These inquiries are pivotal for understanding SARS-CoV-2 transmission dynamics and informing the development of enhanced antiviral strategies to mitigate disease spread. In this study, we leverage a SARS-CoV-2 ferret infection model to provide mechanistic insights into these critical questions.

## MATERIALS AND METHODS

### Facility and biosafety statement

All experiments involving infectious SARS-CoV-2 were conducted in biosafety level 4 (BSL-4) and animal biosafety level 4 (ABSL-4) facilities at the Harbin Veterinary Research Institute (HVRI), Chinese Academy of Agricultural Sciences (CAAS). These facilities are officially approved for high-containment virology research by the Ministry of Agriculture and Rural Affairs of the People’s Republic of China.

### Cells and viruses

Vero E6 cells were maintained in Dulbecco’s modified Eagle’s medium (DMEM) supplemented with 10% fetal bovine serum (FBS) and antibiotics, and cultured at 37°C in a 5% CO_2_ incubator. The SARS-CoV-2 strain HRB25 (GISAID accession no. EPI_ISL_467430; full designation: SARS-CoV-2/HRB25/human/2020/CHN) was isolated from a patient and propagated in Vero E6 cells. Viral stocks were generated in Vero E6 cells using DMEM containing 5% FBS. A low-passage virus (passage 3) was utilized in the experiments. Furthermore, sequencing and alignment analyses were conducted to confirm the absence of mutations in the viral stock. Infectious virus titers were determined by plaque-forming unit (PFU) assay in Vero E6 cells.

### Quantitative PCR

Viral genomic RNA of SARS-CoV-2 was extracted using the QIAamp Viral RNA Minikit (Qiagen, Hilden, Germany). Reverse transcription for qPCR was performed with the HiScript II Q RT SuperMix (Vazyme, Nanjing, China). Quantitative PCR (qPCR) was conducted to quantify viral *N* gene RNA copies using the Applied Biosystems QuantStudio 5 Real-Time PCR System (Thermo Fisher Scientific, Waltham, MA, USA) with Premix Ex Taq (Probe qPCR) (Takara, Dalian, China). The *N* gene-specific primers (forward: 5′-GGGGAACTTCTCCTGCTAGAAT-3′; reverse: 5′-CAGACATTTTGCTCTCAAGCTG-3′) and probe (5′-FAM-TTGCTGCTGCTTGACAGATT-TAMRA-3′) were designed according to the protocols provided by the National Institute for Viral Disease Control and Prevention, China (http://nmdc.cn/nCoV). The quantity of vRNA for the target SARS-CoV-2 *N* gene was normalized against a standard curve generated using a plasmid (pBluescriptII SK-N, 4,221 bp) containing the full-length cDNA of the SARS-CoV-2 *N* gene.

### Enzyme linked immunosorbent assay

Antibodies against SARS-CoV-2 were detected using a Double Antigen Sandwich ELISA Kit (ProtTech, Luoyang, China) following the manufacturer’s instructions. Briefly, 100 µL of serum samples was added to antigen-precoated microtiter plates and incubated at 37°C for 30 min. The plates were then washed five times with PBST (phosphate-buffered saline with Tween 20) and incubated with HRP-conjugated antigen at 37°C for 30 min. After five additional PBST washes, 100 µL of substrate solution was added to initiate color development. The reaction was terminated by adding 50 µL of stop buffer, and the optical density (OD) was measured at 450 nm.

### Plaque reduction neutralization assays

The SARS-CoV-2 HRB-25 strain (50 PFU) was incubated with twofold serial dilutions of serum samples at 37°C for 1 h. Plaque reduction neutralization (PRN) assays were subsequently performed in Vero E6 cells using the virus-serum neutralization mixtures. Neutralizing antibody titers were defined as the highest serum dilution that resulted in a 50% reduction in plaque number compared to the control serum derived from uninfected animals.

### Vaccine and immunization

Recombinant adenovirus expressing the SARS-CoV-2 HRB25 spike (S) protein was generated using the AdEasy XL Adenoviral Vector System (Stratagene, USA). Briefly, the mammalian codon-optimized *S* gene of HRB25, with its native signal peptide replaced by that of tissue plasminogen activator (tPA), was cloned into the pShuttle-CMV vector. Following homologous recombination with the pAdEasy-1 vector in *Escherichia coli*, the linearized recombinant adenoviral plasmid was transfected into AD-293 cells to rescue infectious recombinant adenovirus. The resultant virus, designated rAd-S, was propagated, titrated, and aliquoted for storage until use.

Three groups of 3- to 4-month-old female ferrets (*n* = 6 per group) were immunized with a single dose of the rAd-S vaccine via intramuscular (IM), intranasal (IN), or oral routes. Each ferret received a 1 mL volume containing 1 × 10^9^ TCID_50_/mL of the vaccine. Five and seven weeks post-immunization, three immunized ferrets (recipients) in each group were co-housed with three SARS-CoV-2-infected donor ferrets to assess viral transmission. S protein-specific IgG and SARS-CoV-2 neutralizing antibodies in immunized ferrets were quantified prior to co-housing.

### Ferret study

All ferrets tested serologically negative for SARS-CoV-2 prior to experiment initiation. Three- to four-month-old female ferrets (Wuxi Cay Ferret Farm, Wuxi, China) were used in this study. For the ferret infection assay, two groups of ferrets (*n* = 3 per group) were intranasally inoculated with 1 × 10^7^ PFU or 1 × 10^6^ PFU of the HRB25 strain per ferret, respectively. Ferrets were monitored daily for clinical signs of illness. Nasal washes were collected at 2-day intervals for 12 consecutive days to quantify viral RNA copies and infectious viruses. Convalescent sera were collected to assay for S protein-specific IgG and neutralizing antibodies. For the direct contact transmission study, groups of three ferrets were anesthetized with Zoletil 50 (Virbac, Carros, France) and intranasally inoculated with 1 × 10^7^ PFU of SARS-CoV-2 HRB25 in a 1 mL volume, then co-housed in a single cage. Three naive ferrets were introduced into the same cage 24 hours post-infection (p.i.). Nasal washes were collected at 2-day intervals for viral RNA and infectious virus detection. All ferrets were humanely euthanized on day 19 p.i., and sera were collected to analyze S protein-specific IgG and neutralizing antibodies.

### Statistical analysis

The analyses were conducted using GraphPad Prism v.8.0.2.

## RESULTS

### SARS-CoV-2 transmits efficiently between ferrets via close contact

**Fig 1:**
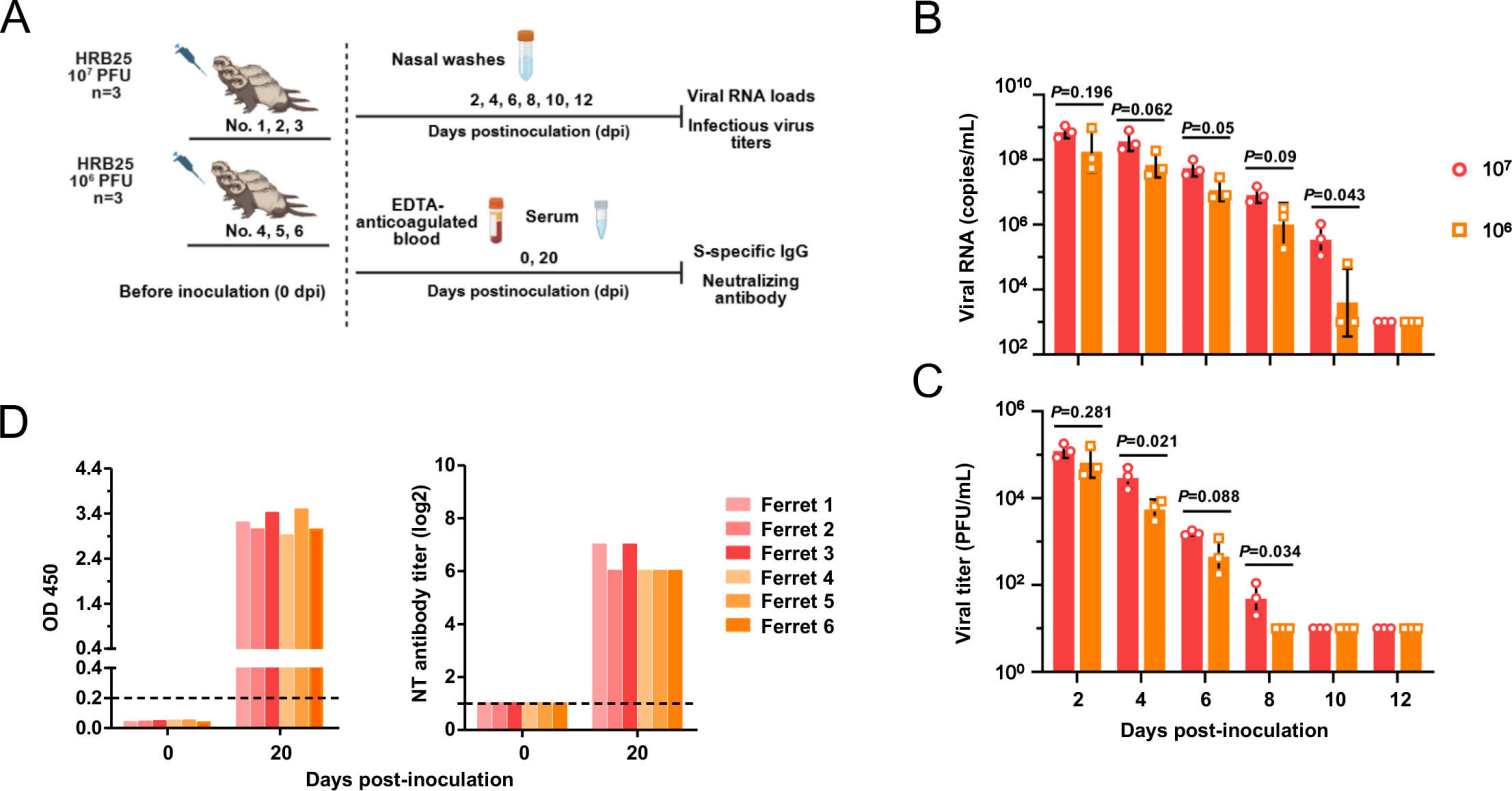
Replication of HRB25 in ferrets. Naive ferrets were intranasally inoculated with 1 × 10^7^ (ferret 1, 2, and 3) or 1 × 10^6^ (ferret 4, 5, and 6) PFU HRB25 (**A**). Viral RNA loads (**B**) and infectious virus titers (**C**) in nasal washes were quantified at two-day intervals for 12 days post-inoculation. Serum samples collected from ferrets at pre-inoculation and 20 days post-inoculation were analyzed for the presence of S protein-specific Ig and neutralizing antibodies (**D**). Statistical significance was determined using the Holm-Sidak multiple-comparison *t*-test, with alpha = 0.05.

To characterize SARS-CoV-2 transmission dynamics, we first investigated the infection kinetics and viral shedding of the HRB25 strain in ferrets (Fig. 1A). Results demonstrated that ferrets are highly susceptible to SARS-CoV-2 infection, with the virus establishing rapid infection and efficient replication in the nasal turbinate. Viral shedding was detected in nasal washes, peaking at day 2 p.i. Viral RNA remained detectable until day 10 p.i., while infectious virus was recoverable until day 8 p.i. No statistically significant differences in viral RNA loads or infectious virus titers were observed between ferrets inoculated with 1 × 10^7^ vs 1 × 10^6^ PFU (Fig. 1B and C). All ferrets in the challenge groups had developed S-specific serum IgG antibodies and neutralizing antibodies by 20 days post-inoculation (Fig. 1D). These antibodies were undetectable in the ferrets prior to the challenge; however, a significant increase in antibody levels was observed by day 20 post-challenge. This indicates that ferrets recovered from SARS-CoV-2 infection are capable of generating SARS-CoV-2-specific antibodies.

**Fig 2:**
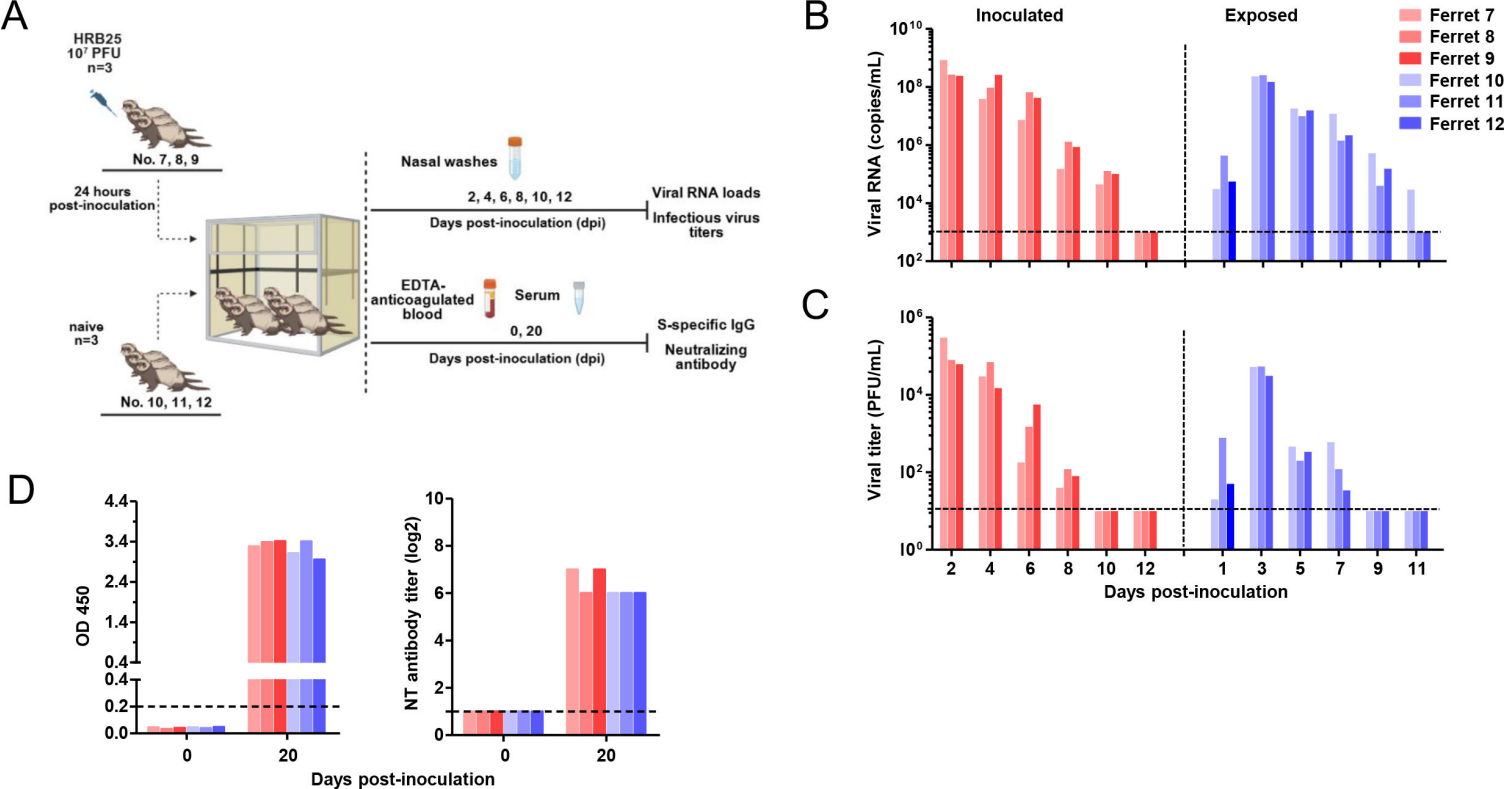
Transmission of SARS-CoV-2 between co-housed naive ferrets. Donor ferrets (ferret 7, 8, and 9) were intranasally inoculated with 1 × 10^7^ PFU HRB25. Recipient ferrets (ferret 10, 11, and 12) were co-housed with the donor ferrets 24 h post-inoculation (**A**). Viral RNA loads (**B**) and infectious virus titers (**C**) in nasal washes were quantified at specified time points. Serum samples collected from ferrets at pre-inoculation, and 20 days post-inoculation were analyzed for the presence of S protein-specific Ig and neutralizing antibodies (**D**).

Ferrets are widely used as an animal model for studying the pathogenicity and transmissibility of respiratory viruses (9, 10). Consistent with previous reports, our data confirmed ferrets exhibit high susceptibility to SARS-CoV-2 (2, 11, 12). To assess viral replication, shedding, and transmissibility, nine 3- to 4-month-old female ferrets were intranasally inoculated with 1 × 10^7^ PFU of HRB25 in a 1 mL volume. Groups of three inoculated ferrets were co-housed in cages for direct transmission studies. Twenty-four hours post-inoculation, three age-matched naive ferrets were introduced into one of the inoculated groups for direct contact transmission analysis. Nasal washes were collected on days 2, 4, 6, 8, 10, and 12 p.i. for viral RNA quantification by qPCR and infectious virus titration by PFU assay (Fig. 2A).

Results showed viral RNA loads in inoculated ferrets peaked at day 2 p.i. and persisted until day 8–10 p.i., with infectious virus isolated up to day 6–8 p.i. In direct contact recipient ferrets, viral RNA and infectious virus were detected in nasal washes as early as day 2 p.i. (1 day post-contact), with titers peaking at day 4 p.i. (3 days post-contact) (Fig. 2B and C). Recovered ferrets exhibited robust induction of S-protein-specific IgG and neutralizing antibodies (Fig. 2D). Collectively, these data demonstrate that the SARS-CoV-2 HRB25 strain is efficiently transmitted among naive ferrets via direct contact. All convalescent ferrets developed SARS-CoV-2-specific humoral immunity, as evidenced by detectable IgG and neutralizing antibodies.

### Convalescent ferrets have lowered susceptibility to SARS-CoV-2 re-infection as recipient, and they are not likely transmitting virus as donors within 7 weeks post initial infection

**Fig 3:**
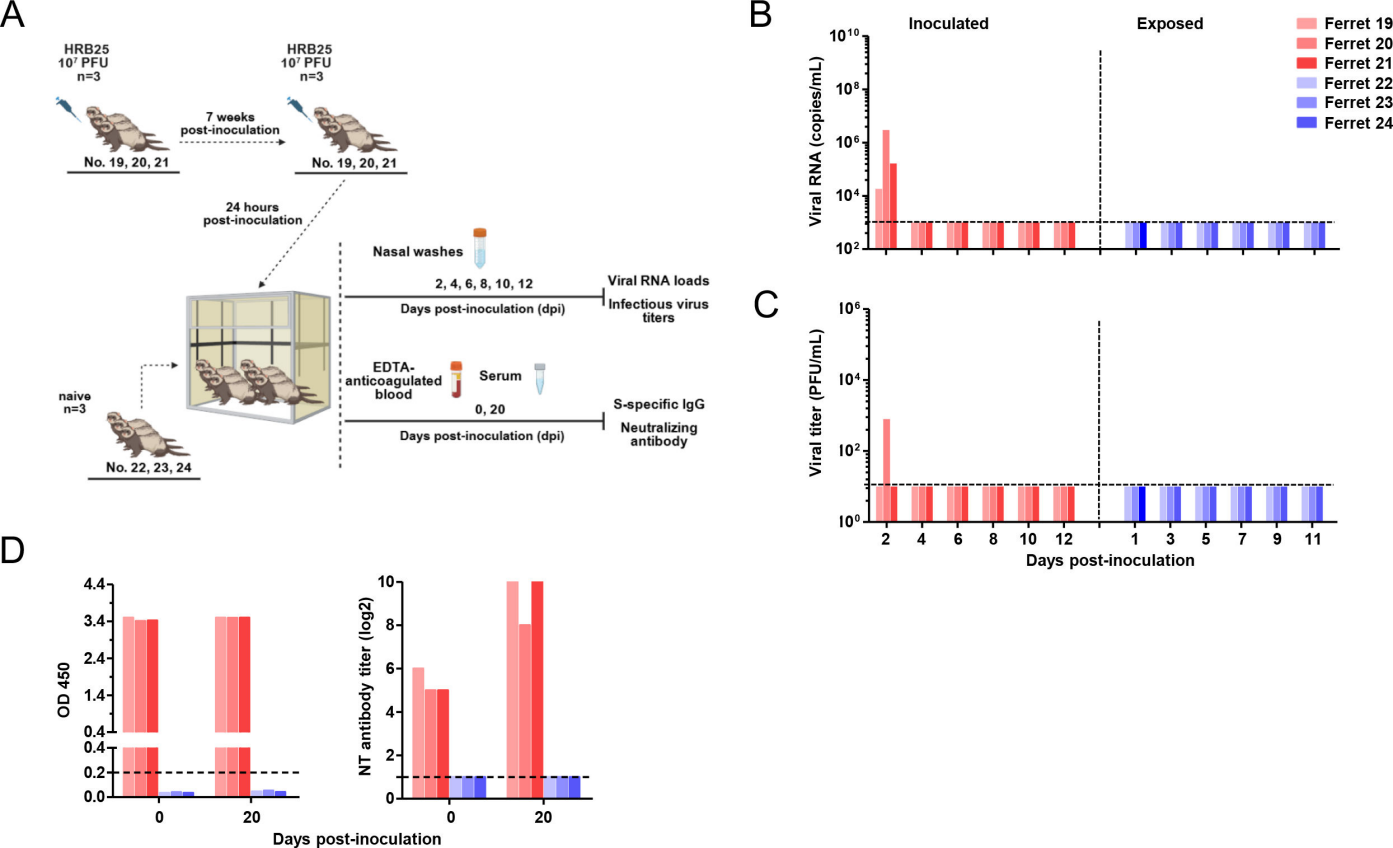
Transmission of SARS-CoV-2 between co-housed convalescent donor ferrets and naive recipient ferrets. Convalescent ferrets (ferret 19, 20, and 21) were intranasally inoculated with 1 × 10^7^ PFU HRB25 seven weeks after initial infection. Recipient ferrets (ferret 22, 23, and 24) were co-housed with the donor ferrets 24 h post-re-inoculation (**A**). Viral RNA loads (**B**) and infectious virus titers (**C**) in nasal washes were quantified at specified time points. Serum samples were collected from ferrets at pre-re-inoculation and 20 days post-re-inoculation to analyze S protein-specific Ig and neutralizing antibodies (**D**).

Occasional reports have described SARS-CoV-2 re-positivity in humans following recovery from infection. To determine whether previously infected and recovered ferrets could be re-infected with SARS-CoV-2, three convalescent ferrets were intranasally re-inoculated with the same dose of HRB25 at 7 weeks post initial infection (p.i.i.). Twenty-four hours later, three age-matched naive ferrets were co-housed with the re-inoculated group to assess transmission risk from re-infected donors (Fig. 3A). At 7 weeks p.i.i., recovered ferrets retained high levels of S protein-specific IgG though neutralizing antibody titers decreased by two- to fourfold compared to day 19 p.i.i.. Upon experimental re-inoculation, infectious virus was detected in nasal washes of 1 out of 3 re-inoculated ferrets at day 2 post re-inoculation (p.r.i.), while all three showed positive viral RNA detection (Fig. 3B and C). However, both viral RNA loads and infectious virus titers were significantly lower than those observed at day 2 p.i.i. during primary infection, with no detectable virus shedding in re-inoculated animals thereafter. Direct contact-naive ferrets remained negative for viral RNA and antibodies throughout the study (Fig. 3B through D). These findings indicate that experimentally re-inoculated ferrets exhibit transient, low-level SARS-CoV-2 replication but are unlikely to transmit virus to naive contacts.

**Fig 4:**
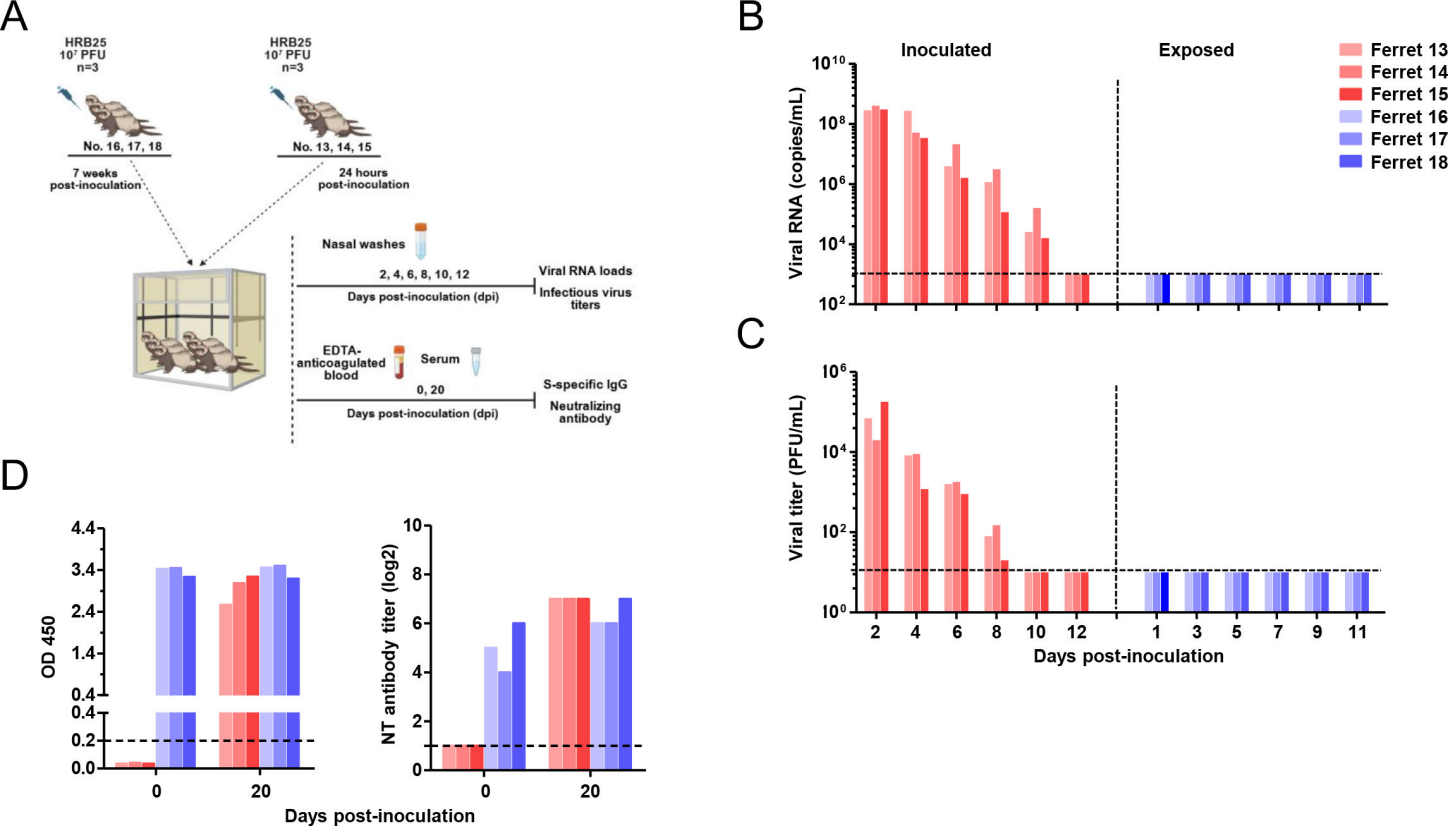
Transmission of SARS-CoV-2 between co-housed naive donor ferrets and convalescent recipient ferrets. Naive donor ferrets (ferret 13, 14, and 15) were intranasally inoculated with 1 × 10^7^ PFU HRB25. Seven weeks after infection, convalescent recipient ferrets (ferret 16, 17, and 18) were co-housed with the donor ferrets 24 hours post-inoculation (**A**). Viral RNA loads (**B**) and infectious virus titers (**C**) in nasal washes were quantified at specified time points. Serum samples were collected from ferrets at pre-inoculation and 20 days post-inoculation to analyze S protein-specific Ig and neutralizing antibodies (**D**). Statistical significance was determined using the Holm-Sidak multiple-comparison *t*-test, with alpha = 0.05.

To evaluate re-infection risk via continuous exposure to shedding viruses, three naive ferrets were inoculated with 1 × 10^7^ PFU of HRB25, and 24 hours later, three recovered ferrets (7 weeks p.i.i.) were co-housed as recipients (Fig. 4A). Inoculated donors displayed viral shedding kinetics comparable to prior experiments (Fig. 1 and 2), but no viral RNA or infectious virus was detected in the nasal washes of contact-exposed recovered ferrets (Fig. 4B and C). These data demonstrate that convalescent ferrets are resistant to re-infection via naturalistic virus exposure for at least 7 weeks p.i.i.. We collected blood samples from all ferrets prior to the challenge and on day 20 post-challenge. Serum levels of S protein-specific IgG antibodies were quantified via ELISA, while neutralizing antibodies in serum were measured using virus neutralization assays. The results demonstrated that ferrets undergoing the primary challenge had seroconverted for S-specific IgG antibodies and neutralizing antibodies by day 20 post-challenge. In contrast, for ferrets that had recovered from the infection (7 weeks after the primary challenge), no significant changes were detected in the levels of serum S protein-specific IgG antibodies following direct contact with donor ferrets, whereas a slight elevation was observed in their neutralizing antibody levels (Fig. 4D).

**Fig 5:**
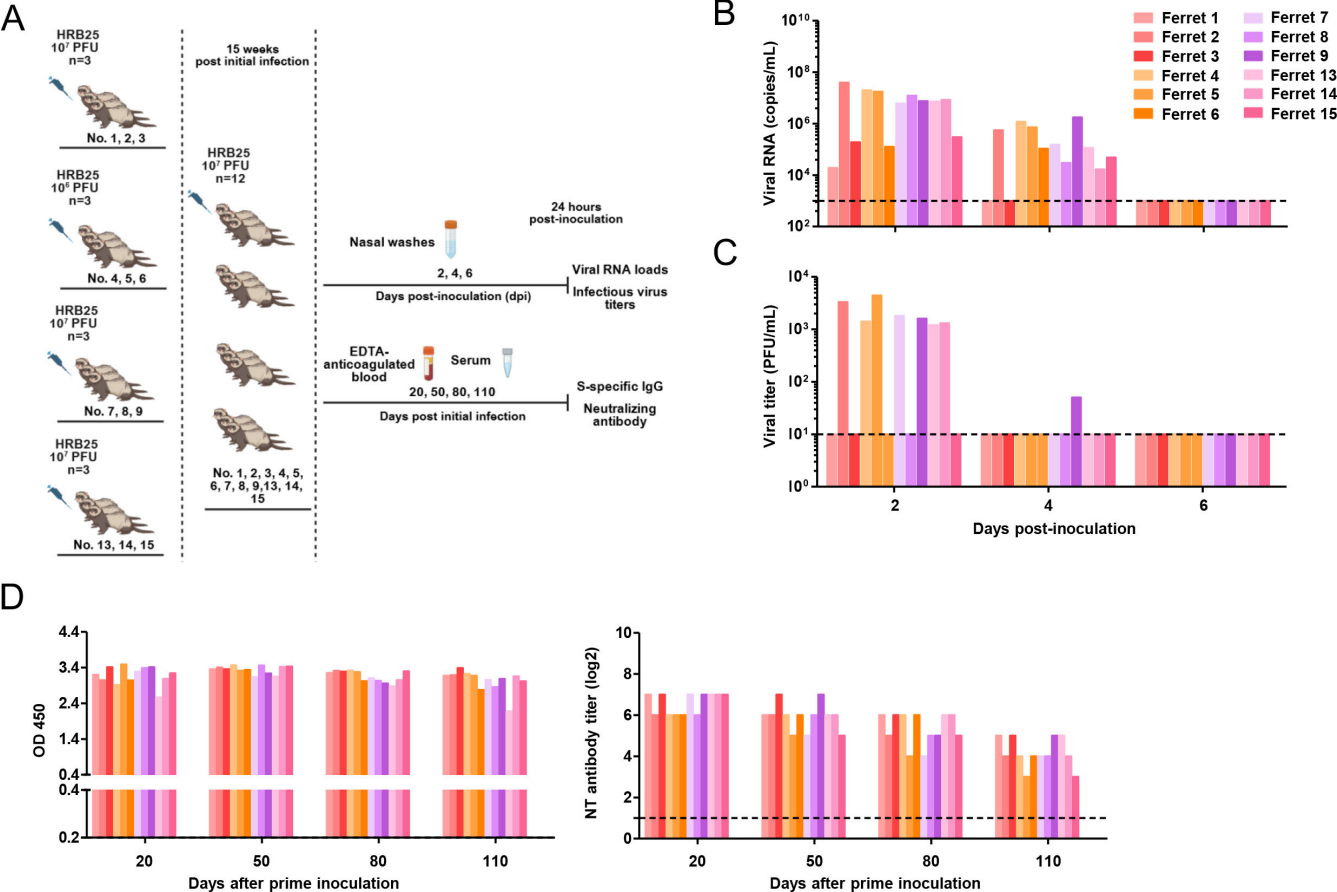
Virus shedding of convalescent ferrets challenged at 15 weeks post-initial infection. Convalescent ferrets (ferret 1–9, 13–15) were re-inoculated with 1 × 10^7^ PFU HRB25 15 weeks after their initial infection (**A**). Viral RNA loads (**B**) and infectious virus titers (**C**) in nasal washes were quantified at specified time points. Serum samples were collected from ferrets at 20, 50, 80, and 110 days post-inoculation to analyze S protein-specific Ig and neutralizing antibodies (**D**).

To investigate whether prolonged post-infection intervals reduce immunity, 12 convalescent ferrets were inoculated with 10^7^ PFU of HRB25 at 15 weeks p.i.i. (Fig. 5A). Viral RNA was detected in all animals, peaking at day 2 p.i.; 10 ferrets remained vRNA-positive at day 4 p.i., with all testing negative by day 6 p.i.. For infectious virus, 7/12 ferrets tested positive at day 2 p.i., dropping to 1/12 at day 4 p.i., and none by day 6 p.i. (Fig. 5B and C). While convalescent ferrets exhibited lower infection prevalence and shorter shedding duration compared to naive controls, these results indicate that sterile immunity wanes by 15 weeks p.i.i., allowing SARS-CoV-2 re-infection despite detectable neutralizing antibodies (Fig. 5D). Immunological memory responses likely contribute to accelerated viral clearance.

Collectively, these data show that experimentally re-inoculated ferrets can experience transient SARS-CoV-2 replication following high-dose challenge but do not transmit virus to naive contacts. Recovered ferrets resist naturalistic reinfection within 7 weeks p.i.i. but become partially susceptible by 15 weeks p.i.i., with immune memory still modulating viral kinetics. These findings provide critical insights into the durability of natural immunity and re-infection risks in SARS-CoV-2 recovered hosts.

### Susceptibility of rAd-S vaccinated recipient ferrets to SARS-CoV-2 infection via close contact

**Fig 6:**
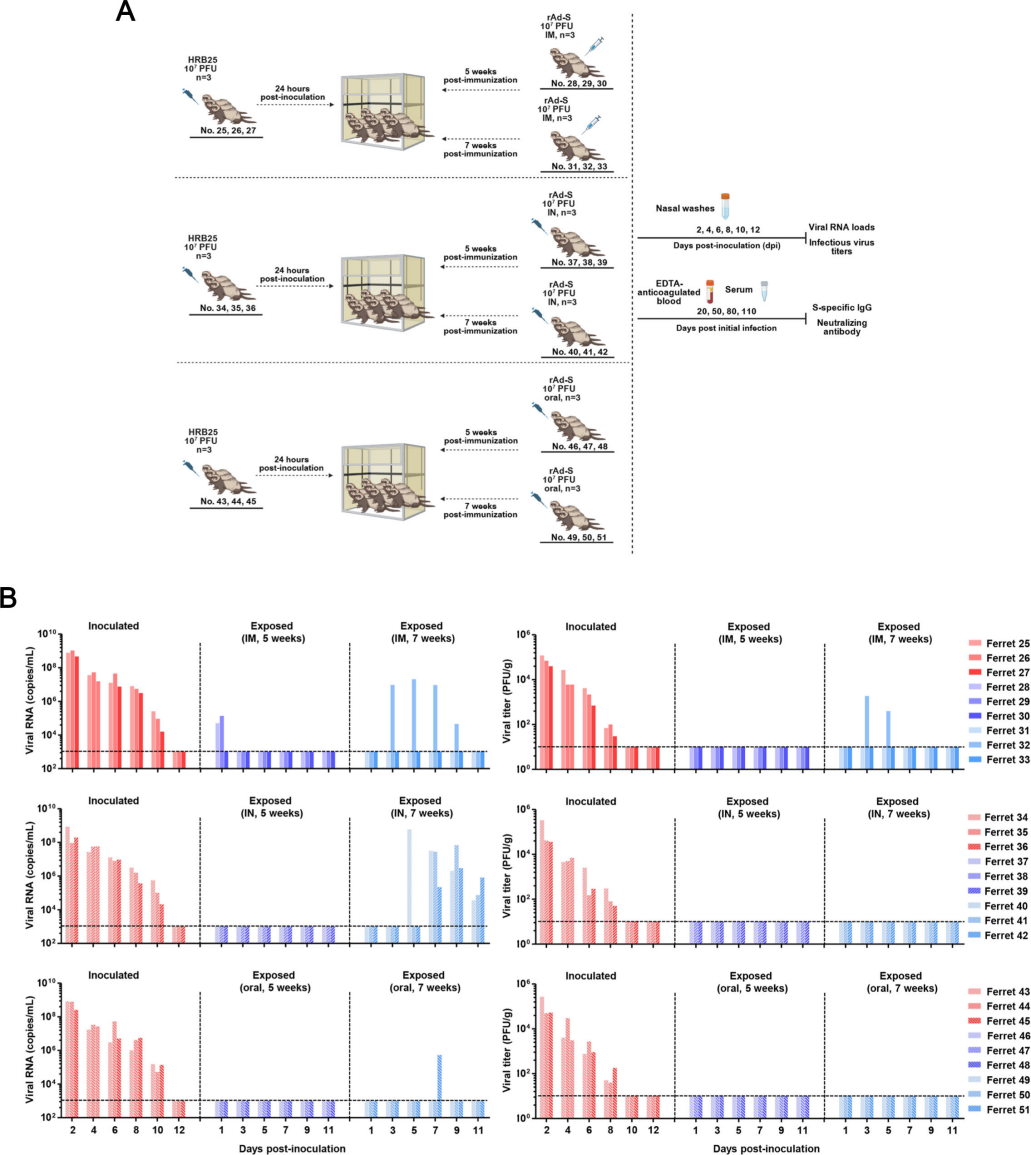
Transmission of SARS-CoV-2 from naive donor ferrets to rAd-S immunized recipient ferrets. Naive donor ferrets (ferret 25–27, 34–36, and 43–45) were intranasally inoculated with 1 × 10^7^ PFU HRB25. Twenty-four hours post-inoculation, recipient ferrets (ferret 28–33, 37–42, and 46–51) previously immunized with a single dose of rAd-S vaccine were co-housed with the donor ferrets to assess viral transmission (**A**). Viral RNA loads and infectious virus titers in nasal washes were quantified at specified time points (**B**).

**Fig 7:**
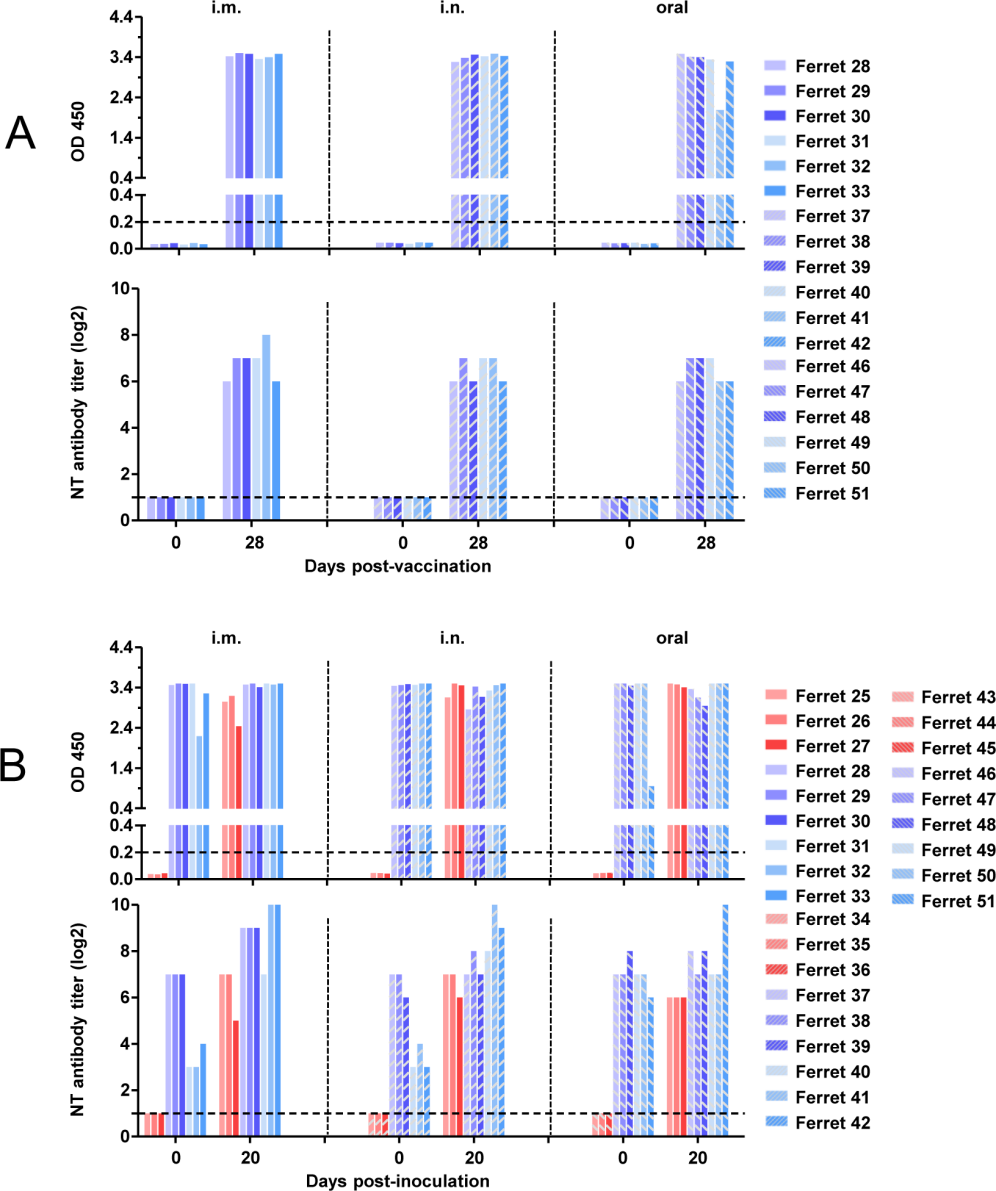
Antibody responses in rAd-S immunized ferrets before and after co-housing with naive donor ferrets. Serum samples were collected from rAd-S immunized ferrets (ferret 28–33, 37–42, and 46–51) at 28 days post-vaccination to analyze S protein-specific Ig and neutralizing antibodies (**A**). Prior and following co-housing, serum samples were collected from ferrets (ferret 25–51), and levels of S protein-specific Ig and neutralizing antibodies were quantified (**B**).

To evaluate the infection risk of vaccinated individuals under natural exposure conditions, we used ferrets vaccinated with a recombinant human adenovirus type 5 (Ad5)-vectored SARS-CoV-2 S protein (rAd-S) as a model. Ferrets were immunized with a single dose of 1 × 10^9^ TCID_50_ per ferret via intramuscular (IM), intranasal (IN), or oral routes. Seroconversion and the presence of neutralizing antibodies were confirmed in all immunized ferrets (Fig. 7). Groups of naive ferrets (*n* = 3) intranasally inoculated with 1 × 10^7^ PFU of HRB25 served as virus donors. Twenty-four hours post-inoculation, immunized ferrets (5 and 7 weeks post-immunization) were co-housed with donor ferrets for transmission assays (Fig. 6A).

Ferrets immunized 5 weeks prior showed robust resistance to donor-derived virus. No viral RNA or infectious virus was detected in nasal washes of IN- or orally immunized ferrets throughout the experiment. Among IM-immunized ferrets, only 2/3 exhibited transient low-level viral RNA in nasal washes at day 2 post-infection (p.i.), with no detectable infectious virus (Fig. 6B, upper panel).

By 7 weeks post-immunization, viral RNA was detected in some ferrets across all immunization groups during co-housing. Infectious virus remained undetectable in IN- and orally immunized ferrets, except for one IM-immunized ferret that tested positive for infectious virus on days 3 and 5 post-co-housing (Fig. 6B).

Following co-housing of immunized ferrets with donor ferrets, antibodies were assessed in all animals. Among these, immunized ferrets that became infected after exposure to infected donor ferrets included Ferrets 28, 29, 32, 40, 41, 42, and 51. Comparison with results in Fig. 7 revealed that between day 28 post-immunization and the initiation of co-housing with donor ferrets: Ferret 32 exhibited a reduction in S-specific IgG, with neutralizing antibody titers decreasing from 2^8^ to 2^3^; Ferret 40 showed no change in S-specific IgG, while neutralizing antibody titers declined from 2^7^ to 2^3^; Ferret 41 had stable S-specific IgG levels, with neutralizing antibody titers dropping from 2^7^ to 2^4^; Ferret 42 displayed no alteration in S-specific IgG, while neutralizing antibody titers decreased from 2^6^ to 2^3^; Ferret 51 demonstrated reduced S-specific IgG, with neutralizing antibody titers remaining unchanged. These findings suggest that reinfection in immunized ferrets is primarily due to a decline in virus-specific antibodies.

Collectively, these results demonstrate that a single dose of rAd-S vaccine induces protective immunity against SARS-CoV-2 transmission from virus-shedding donors for at least 5 weeks post-immunization. Susceptibility increased at 7 weeks post-immunization, coinciding with higher viral RNA positivity rates. Notably, infectious virus was rarely detected in nasal washes across all groups, indicating limited virus shedding potential. Mucosal immunization routes (IN and oral) consistently outperformed the intramuscular route in conferring durable resistance to SARS-CoV-2 in ferrets.

## DISCUSSION

Since the outbreak of COVID-19, it has imposed a tremendous social burden and caused significant economic losses. Given the high transmissibility and rapid spread of SARS-CoV-2, the ongoing circulation of its variants has shown no signs of abating to date. The global community has made concerted efforts to accelerate the development of SARS-CoV-2 vaccines and antiviral drugs. Currently, dozens of vaccines and antiviral drugs have been approved for use. Vaccination is expected to alter the transmission dynamics of SARS-CoV-2, which raises a series of research questions deserving in-depth investigation. For instance: How efficiently and rapidly can the virus still be transmitted? Can the pre-existing immunity in convalescent patients or vaccine recipients effectively prevent re-infection and viral shedding? These questions are pivotal to elucidating the transmission patterns of SARS-CoV-2 and to providing critical scientific evidence for formulating targeted antiviral strategies—ultimately mitigating the ongoing circulation of its variants.

Ferrets have emerged as a valuable infection model for investigating the transmission dynamics and immunological responses to SARS-CoV-2 (13). Our prior research demonstrated that ferrets are susceptible to SARS-CoV-2 infection, with detectable viral loads in nasal wash samples at significant levels (2). In this study, we further characterized SARS-CoV-2 transmission in ferrets under close-contact conditions. We observed efficient viral transmission between co-housed ferrets: infectious virus was detected in the nasal washes of recipient ferrets as early as 2 days post-co-housing with infected donor ferrets. Notably, all recipient ferrets developed SARS-CoV-2-neutralizing antibody titers comparable to those of the donor ferrets. These findings collectively demonstrate robust viral replication and rapid horizontal transmission of SARS-CoV-2 in ferret co-housing environments. While previous studies (11, 12) reported limited SARS-CoV-2 transmission in ferrets under controlled laboratory conditions with regulated airflow, ferret farms typically involve continuous direct contact among animals. Thus, elucidating transmission patterns in close-contact scenarios is of critical practical importance. Our results highlight that co-housing significantly enhances SARS-CoV-2 transmissibility compared to isolated laboratory settings.

The ongoing circulation of SARS-CoV-2 variants shows no signs of abating, which means a growing number of convalescent patients and vaccine recipients remain continuously vulnerable to (re)exposure. Investigating viral infection in the presence of pre-existing immunity and potential viral shedding is therefore of critical importance. COVID-19 transmission is predominantly characterized by cluster infections, with epidemiological data indicating that respiratory droplets expelled during face-to-face interactions-such as talking, coughing, or sneezing-represent the primary route of spread. Prolonged exposure to an infected person and briefer exposures to individuals who are symptomatic are associated with higher risk for transmission, whereas brief contact with asymptomatic carriers is less likely to result in infection (14). To model these real-world exposure scenarios, we utilized co-housed ferrets. Our results demonstrated that convalescent ferrets (7 weeks p.i.) served as susceptible donors under artificial inoculation, exhibiting transient infection: viral RNA and infectious viruses were detected only at day 2 p.i., with subsequent negative results thereafter. Co-housed naive recipient ferrets remained uninfected, as evidenced by negative tests for viral RNA, infectious viruses in nasal washes, and absence of seroconversion. When convalescent ferrets served as recipients, no viral transmission was observed, with all tests for viral RNA and infectious viruses remaining negative throughout the experiment. Convalescent ferrets re-infected at a prolonged interval (15 weeks p.i.) exhibited higher levels of viral RNA and infectious viruses, delayed viral clearance, and reduced neutralizing antibody titers compared to those re-infected at 7 weeks p.i. These findings indicate that immunity induced by initial infection promotes rapid viral clearance in donors and protects recipients from infection. The risk of re-infection increases over time post-initial infection, primarily due to the decline in neutralizing antibody levels.

Vaccination serves as a validated strategy to prevent SARS-CoV-2 infection, particularly in protecting individuals with immunocompromised status from progressing to severe disease following viral exposure. Current studies have demonstrated the efficacy of several vaccines. Among these, a recombinant adenovirus-vectored SARS-CoV-2 vaccine has shown good tolerability and efficacy in preclinical and clinical evaluations (15, 16). Mucosally immunized ferrets developed sterile immunity against SARS-CoV-2 challenge, with no detectable viral RNA or infectious virus in nasal washes at four weeks post-immunization (13), indicating they do not exhibit viral shedding. To investigate the susceptibility of vaccinated individuals in close-contact transmission scenarios, we evaluated the infection risk of rAd-S-vaccinated ferrets (as recipients) following prolonged exposure to co-housed, virus-shedding donor ferrets at extended time points post-immunization. Ferrets 5 weeks post-immunization exhibited complete protection against infection: no infectious virus was detected in nasal washes of any ferret, directly demonstrating that vaccination blocked viral transmission. In the ferret cohort at 7 weeks post-immunization, viral RNA was detected in subset of individuals across the IN-, IM-, and oral immunization groups. Infectious virus remained undetected in the IN- and oral immunization groups, with the sole exception of one ferret in the IM-immunization group. These findings indicate that immunity remains functional but began to wane at this time point, largely attributed to declining neutralizing antibody titers. Collectively, these results indicate that mucosal immunization (IN/oral routes) may confer superior and more durable protective immunity against SARS-CoV-2 compared to IM-immunization. Thus, vaccinated individuals may require periodic monitoring of neutralizing antibody levels and receive booster vaccinations when necessary.

## Data Availability

The data supporting the findings of this study are available within the article.
